# Genome-Wide Transcriptome Profiling Revealed Cotton Fuzz Fiber Development Having a Similar Molecular Model as *Arabidopsis* Trichome

**DOI:** 10.1371/journal.pone.0097313

**Published:** 2014-05-13

**Authors:** Qun Wan, Hua Zhang, Wenxue Ye, Huaitong Wu, Tianzhen Zhang

**Affiliations:** National Key Laboratory of Crop Genetics and Germplasm Enhancement, Cotton Research Institute, Nanjing Agricultural University, Nanjing, China; University of Massachusetts Amherst, United States of America

## Abstract

The cotton fiber, as a single-celled trichome, is a biological model system for studying cell differentiation and elongation. However, the complexity of gene expression and regulation in the fiber complicates genetic research. In this study, we investigated the genome-wide transcriptome profiling in Texas Marker-1 (TM-1) and five naked seed or fuzzless mutants (three dominant and two recessive) during the fuzz initial development stage. More than three million clean tags were generated from each sample representing the expression data for 27,325 genes, which account for 72.8% of the annotated *Gossypium raimondii* primary transcript genes. Thousands of differentially expressed genes (DEGs) were identified between TM-1 and the mutants. Based on functional enrichment analysis, the DEGs downregulated in the mutants were enriched in protein synthesis-related genes and transcription factors, while DEGs upregulated in the mutants were enriched in DNA/chromatin structure-related genes and transcription factors. Pathway analysis showed that ATP synthesis, and sugar and lipid metabolism-related pathways play important roles in fuzz initial development. Also, we identified a large number of transcription factors such as MYB, bHLH, HB, WRKY, AP2/EREBP, bZIP and C2H2 zinc finger families that were differently expressed between TM-1 and the mutants, and were also related to trichome development in *Arabidopsis*.

## Introduction

Cotton (*Gossypium* spp.) is an important commercial crop and the largest source of natural textile fibers grown throughout the world. Cotton fibers used in textiles originate from the outer epidermal layer of the maturing seed, and are classified into two types: lint and fuzz. Initiation of lint fibers is a quasi-synchronous process that occurs in developing ovules during anthesis. The fuzz fibers initiate growth at 4 DPA (days post anthesis) and elongate to approximately 0.5 cm, much shorter than lint fibers [1].

Many genes from *Arabidopsis* have been identified that control the initiation and morphogenesis of trichomes, and most of them encode transcription factors including MYB (*GLABROUS1, TRIPTYCHON, CAPRICE, WEREWOLF*) [2]–[5], WD-40 type (*TRANSPARENT TESTA GLABRA1*) [6], [7], bHLH (*GLABROUS3*) [8], [9], HD-ZIP (*GLABROUS2*) [10] and a WRKY-related transcription factor (*TRANSPARENT TESTA GLABRA2*) [11]. *TRANSPARENT TESTA GLABRA 1* (*TTG1*) encodes a small protein with WD-repeats, although no WD-repeat protein has either enzymatic activity or a DNA binding domain [12]. The identification of TTG1 as a WD40 repeat-containing protein suggests that TTG1 regulates MYC transcription factors or pathways in which MYC factors are involved [7]. *TTG2* encodes a WRKY transcription factor and acts downstream of the trichome initiation genes, *TTG1* and *GL1* [11]. bHLH family members have a basic helix–loop–helix domain [13]. Mutant analyses have identified several plant bHLH proteins involved in anthocyanin biosynthesis, such as GL3, EGL3 and TT8 [8], [9], [14]–[16].

Cotton fibers share many similarities with *A. thaliana* leaves trichome development, and several studies have demonstrated a close relationship between these two types of cells using cotton fiber-related genes (Table S1). Six putative cotton MYB genes (*GhMYB1-6*) have been isolated, and these DNA-binding factors were shown to be involved in the differentiation and expansion of cotton seed trichomes [17]. *GhMYB109*, which encodes a R2R3 MYB transcription factor, was shown to be expressed specifically in fiber cell initials and expanding fibers [18]. Another R2R3 MYB gene, *GaMYB2*, which is homologous to *AtGL1*, was predominantly expressed early in cotton fibers and complemented *gl1* phenotypes in *Arabidopsis* [19]. Overexpressing *GhMYB2* or its downstream gene *GhRDL1* in *Arabidopsis* activates fiber-like hair production on 4–6% of the seed coats and has no obvious effect on trichome development in leaves or siliques [20]. In addition, overexpression of *GbMYB2* in *Arabidopsis* caused thicker leaf trichomes and longer roots to develop due to the activation of trichome development-related genes such as *GL2* [21]. *GhMYB25* encodes a homolog of *AmMIXTA/AmMYBML1* which involved in epidermal cell differentiation, is highly expressed in ovules, fiber cell initials and trichomes on leaf. Silencing of *GhMYB25* in cotton showed fiber and trichome development were suppressed, while overexpression of *GhMYB25* increased cotton fibre initiation and leaf trichome number [22]–[26]. *GhMYB25-like* had a similar expression pattern with *GhMYB25* which significantly higher expression during fiber cell initiation (−3∼3 DPA). Transgenic plants showed *GhMYB25-like* had significant regulatory roles in cotton fiber development. RNA interference suppression of *GhMYB25-like* resulted in cotton plants with fibreless seeds, but normal trichomes elsewhere implying *GhMYB25-like* playing a crucial role in the very early stages of fiber cell differentiation [26], [27]. A cotton gene encoding an *Arabidopsis CPC* ortholog (*R3 MYB* gene) was identified and downregulated in fiber initials at 1 DPA [28]. In addition to the MYB genes, four putative homologues of *Arabidopsis TTG1* (*GhTTG1*–*GhTTG4*), have been isolated and were shown to form two groups, with *GhTTG1* and *GhTTG3* being closely related to each other, and *GhTTG2* and *GhTTG4* forming the second group, based on sequence comparisons of the four deduced proteins and *Arabidopsis TTG1* [29]. Three homeobox (HOX) genes, *GhHOX1*, *GhHOX2*, and *GhHOX3*, have been identified from cotton, showing 66%, 34%, and 37% protein sequence similarity to *Arabidopsis* GL2, respectively. *GhHOX1* was able to restore the glabrous phenotype of *gl2* mutant, indicating that this protein is a functional homologue of GL2 in controlling trichome development and may function in fiber development [30]. Two GL3-like bHLH cDNAs from cotton ovule, *GhDEL65* and *GhDEL61*, have been deposited in the Genbank [31], [32]. It will be interesting to examine if they work like GL3 during cotton fiber development. Also, several ESTs (expressed sequence tags) from cotton have been published that share identity with *Arabidopsis* homologues in the NCBI database [33], [34]. As many homologous genes have been isolated from cotton and shown to play similar roles in trichome initiation in *Arabidopsis*, the GL1-GL3/EGL3-TTG1 protein complex may control fiber formation in cotton [33].

Several “qualitative” mutants in fiber development have been reported. The best characterized of these are the naked seed loci, N_1_N_1_ and n_2_n_2_. The dominant naked seed mutant (NSM) N_1_NSM is fuzzless but with a little lint on the seed [35]. The recessive naked seed mutant n_2_NSM produces regular lint, but bears a naked seed phenotype with very little fuzz fibers present at the micropyle tips of the seed [36]. Fuzzless-lintless mutants (FLM) XZ142FLM, MD17FLM and SL1-7-1FLM are all completely without any fiber; SL1-7-1FLM possess the dominant naked seed gene *N_1_* [37], [38], XZ142FLM possess recessive naked seed gene *n_2_* [39], [40], while MD17FLM possess both *N_1_* and *n_2_* [37], [41]. TM-1 with lint and fuzz fiber is upland genetic standard line, which widely used in research programs [42]. Although these six materials have different genetic background, critical genes or pathways can be identified by studying the common different expressed genes between WT and several same genotype mutants.

To gain a better understanding of gene regulation in the early stage of fuzz development, we present here the first genome-wide analysis of gene expression during cotton fuzz initial cell development using massively parallel deep-sequencing developed by Solexa/Illumina. As cell fate determination for fiber (lint and fuzz) must occur prior to the formation of fiber cell initials, we selected +1, +3 and +5 DPA ovules to analyze fuzz initial development. In this study, we annotated thousands of read signatures matching predicted genes, and quantified the transcript abundance in developing ovules and fibers. In addition, we have profiled gene expression in the mutants against fuzz-bearing ovules (wild type, WT), and found large changes in gene expression in the mutants.

## Materials and Methods

### Plant Material Preparation and Total RNA Isolation

**Figure 1:**
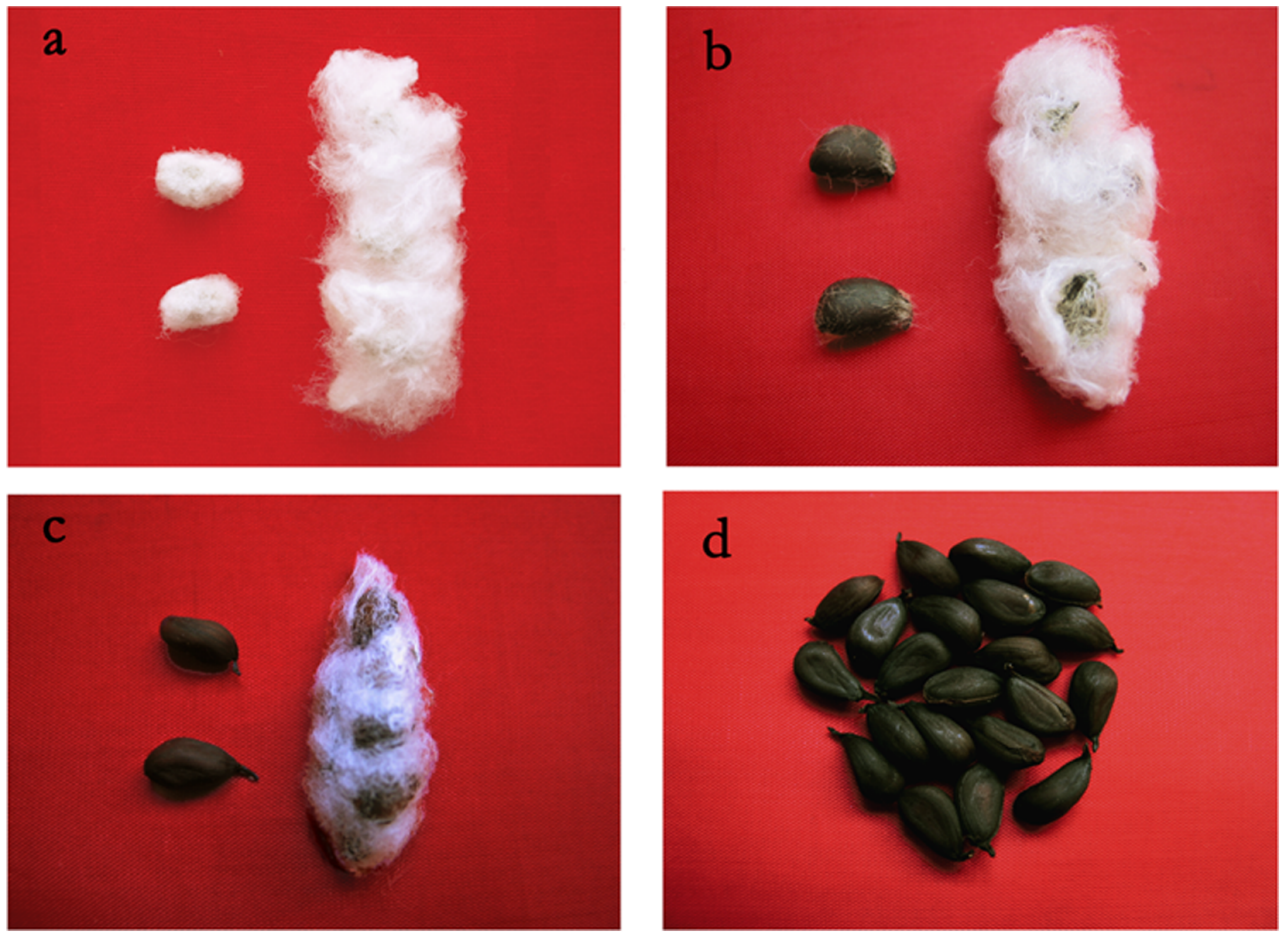
Cotton fiber morphology in the wild-type line and the mutant lines. a: wild line: TM-1; b: recessive naked-seed mutant: n_2_NSM; c: dominant naked-seed mutant: N_1_NSM; d: fuzzless-lintless mutants: SL1-7-1FLM (with *N_1_* gene), XZ142FLM (with *n_2_* gene), MD17FLM (with *N_1_* and *n_2_*). Matured seed were separated from the opened bolls on the cotton plant. Ginned seeds (right) and matured seeds (left) showed on linted-fuzzy and linted-fuzzless panel.

*G. hirsutum* cv. Texas Marker-1 (TM-1) and five naked-seed or fuzzless mutants (XZ142FLM, MD17FLM, SL1-7-1FLM, N_1_NSM and n_2_NSM) were used in this study (Figure 1). SL1-7-1FLM, MD17FLM and N_1_NSM each possess the dominant naked seed gene *N_1_*, while XZ142FLM and n_2_NSM carry the recessive naked seed gene *n_2_*.

Plants were grown at Jiangpu Breeding Station, Nanjing (JBS/NAU) in 2010. All lines were self-pollinated, and the progeny were tested to verify the initial pattern. Buds were tied up the day before anthesis to ensure self-pollination. Bolls were harvested at +1, +3 and +5 DPA. Ovules were excised carefully from bolls, frozen in liquid nitrogen immediately, and stored at −70°C. Total RNA was extracted using the CTAB method [43].

### Sequencing and Digital Tag Profiling

Library construction, sequencing and raw data processing were performed commercially by BGI (Beijing Genomics Institute at Shenzhen, China) *via* the sequencing by synthesis (SBS) on Illumina HiSeq 2000 System as described previously [44]. Digital tag profiling was perfomed as descriped by Wang et al [44] and *Gossypium raimondii* primary transcript sequences (http://www.phytozome.net) was used as reference gene database.

### Defining Differentially Expressed Genes and Cluster Analysis

Statistical analysis was performed to identify differentially expressed genes between the libraries using a rigorous algorithm described previously [45]. Gene expression was normalized to transcripts per million (TPM) clean tags. For gene expression variance, the statistical *t*-test was used to identify genes differently expressed between the libraries. *P* values were adjusted using the multiple testing procedures described by Benjamini and Yekutieli [46] for controlling the false discovery rate (FDR). In this study, we used a stringent value of FDR <0.001, and the absolute value of |log_2_Ratio| ≤1 as the threshold to judge the significant difference of gene expression.

K means clustering was performed with the open-source program Cluster3.0 (http://bonsai.hgc.jp/~mdehoon/software/cluster/software.htm). The genes in each cluster were then classified into Mapman functional categories [47]. Functional categories of the MapMan annotation were tested for significance of expression change by applying a two-sided Wilcoxon rank test with a Benjamini Yekutieli correction for multiple tests. Pathway analysis was mainly based on the Kyoto Encyclopedia of Genes and Genomes (KEGG) database [48].

### Quantitative Real Time RT-PCR (qPCR)

Verification of some differentially expressed genes (DEGs) was performed by real-time quantitative PCR (qPCR). The primers for the various genes were designed with Primer 3.0 (http://frodo.wi.mit.edu/cgi-bin/primer3/primer3), and synthesized commercially (Genscript, Nanjing, China); sequences are given in Table S10. Two microgramme total RNA was reversely transcripted using PrimeScript RT reagent Kit with gDNA Eraser (Perfect Real Time) (TaKaRa, Shiga, Japan). QPCR was performed using the LightCycler FastStart DNA Master SYBR Green I kit (Roche, Basel, Switzerland) in an ABI7500 Real-Time PCR detection system (Applied Biosystems, San Francisco, CA, USA). Each sample was PCR-amplified using 100ng cDNA template in triple reactions. The cotton *histone 3* gene [19] (ACC No. AF024716) was used as the positive control and amplified with the primer pair (F: 5′-GGTGGTGTGAAGAAGCCTCAT-3′, and R: 5′-AATTTCACGAACAAGCCTCTGGAA-3′). The amplification efficiency of each gene was calculated. The qRT-PCR cycles were as follows: (1) 95°C, 10 min; (2) 40 cycles of 95°C for 15 s, ∼60°C (temperature varied for different primers, Table S10) for 30 s and 72°C for 30 s; (3) a melting curve analysis from 65 to 95°C (1 s hold per 0.2°C increase) to check the specificity of the amplified product. Relative expression levels were determined by the 2^−ΔCt^ method.

## Results

### Sequencing Data Analysis

To obtain a global view of transcription relevant to cotton fuzz development, we used the Illumina HiSeq 2000 System to perform high-throughput tag-sequencing (Tag-seq) analysis on poly(A)-enriched RNAs from eighteen cotton ovule libraries including the cultivar TM-1 and five mutants during the fuzz initiation stage (+1 DPA, +3 DPA and +5 DPA). The total number of tags per library ranged from 3.5 to 4.7 million, and the number of tags with distinct sequences ranged from 0.27 to 0.44 million (Table S2). After removal of low quality tags, we obtained a total of 3.4 to 4.5 million clean tags that corresponded to about 0.15 million distinct tags (Table S2). The distribution of total and distinct tag counts over different tag abundance categories showed very similar profiles for all libraries (Figure S1). Among the distinct tags, less than 5% had a copy number higher than 100, whereas 38% of the tags were present between 5 and 50 copies, and more than 57% of the transcripts had 2–5 copies.

As there was no allotetraploid cotton genome sequence available, clean tags were mapped to *G. raimondii* genome sequence (http://www.phytozome.net). Approximately 73%–82% of the distinct tags (83–87% of the total tags) could be mapped to the reference genome (Table S2). All clean tags were aligned to the reference *G. raimondii* primary transcript sequences. Approximately 26%–35% of the distinct tags could be uniquely mapped to the reference sequence. The tags that mapped to the database generated 19,829–22,213 tag-mapped transcripts for the libraries (Table S2).

### Common DEGs between Dominant Naked-seed Mutants and TM-1 during Fuzz Development

To understand the molecular mechanisms of the dominant fuzzy phynotype, 4,358 common DEGs differentially expressed between the mutants MD17FLM, SL1-7-1FLM, N_1_NSM and the wild-type TM-1 were identified. Of these, 268 genes were up regulated and 557 genes down regulated at +1 DPA; 792 genes were up regulated and 699 genes down regulated at +3 DPA in the mutants; and 2,000 genes were up regulated with 957 genes down regulated at +5 DPA. Ten common differentially up regulated genes and 62 down regulated genes were identified at +1 DPA, +3 DPA and +5 DPA (Figure S2, Table S3).

**Figure 2:**
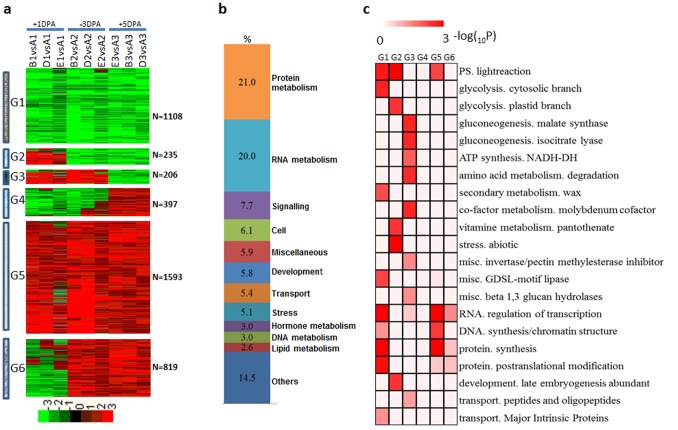
Dynamic progression of common differentially expressed genes in the dominant mutants. (a) Unsupervised hierarchical clustering of the 4,358 common DEGs in the dominant mutants. Common DEGs were clustered into six groups and the number of genes of each group was listed at right. Red region, genes upregulated in the mutants; green region, genes downregulated in the mutants. A, TM-1; B, SL1-7-1FLM; D, MD17FLM; E, N_1_NSM; 1, +1 DPA; 2, +3 DPA; 3, +5 DPA. (b) Functional distribution of common DEGs in the dominant mutants. (c) Functional category enrichment of common differentially expressed genes in the dominant mutants.

We then used MapMan annotation to assign genes to functional categories and grouped the genes into six groups using the hierarchical clustering algorithm. Two main groups (Groups 1 and 5) accounted for ∼62% of the DEGs at the three sampling times (Figure 2a). Excluding 866 genes belonging to the ‘not assigned or unknown’ categories, 3,492 genes had MapMan annotation assignments. Among these, 21.0% are related to protein metabolism, 20.0% to RNA metabolism, 7.7% to signaling, and the remaining genes to cell functions, development, transport, stress, hormone metabolism, DNA metabolism, or lipid metabolism (Figure 2b). To further explore this dataset, we tested for enrichment by MapMan functional category using Fisher’s exact test (*P*<0.01, FDR = 5%). Most of the MapMan bins showed enrichment for particular groups of expressed genes (Figure 2c); for example, genes that encode enzymes for protein synthesis and transcription factors in Group 1, light reaction and abiotic stress in Group 2, ATP synthase, amino acid metabolism, glyoxylate cycle in Group 3, and transcription factors and DNA synthesis in Group 5.

**Figure 3:**
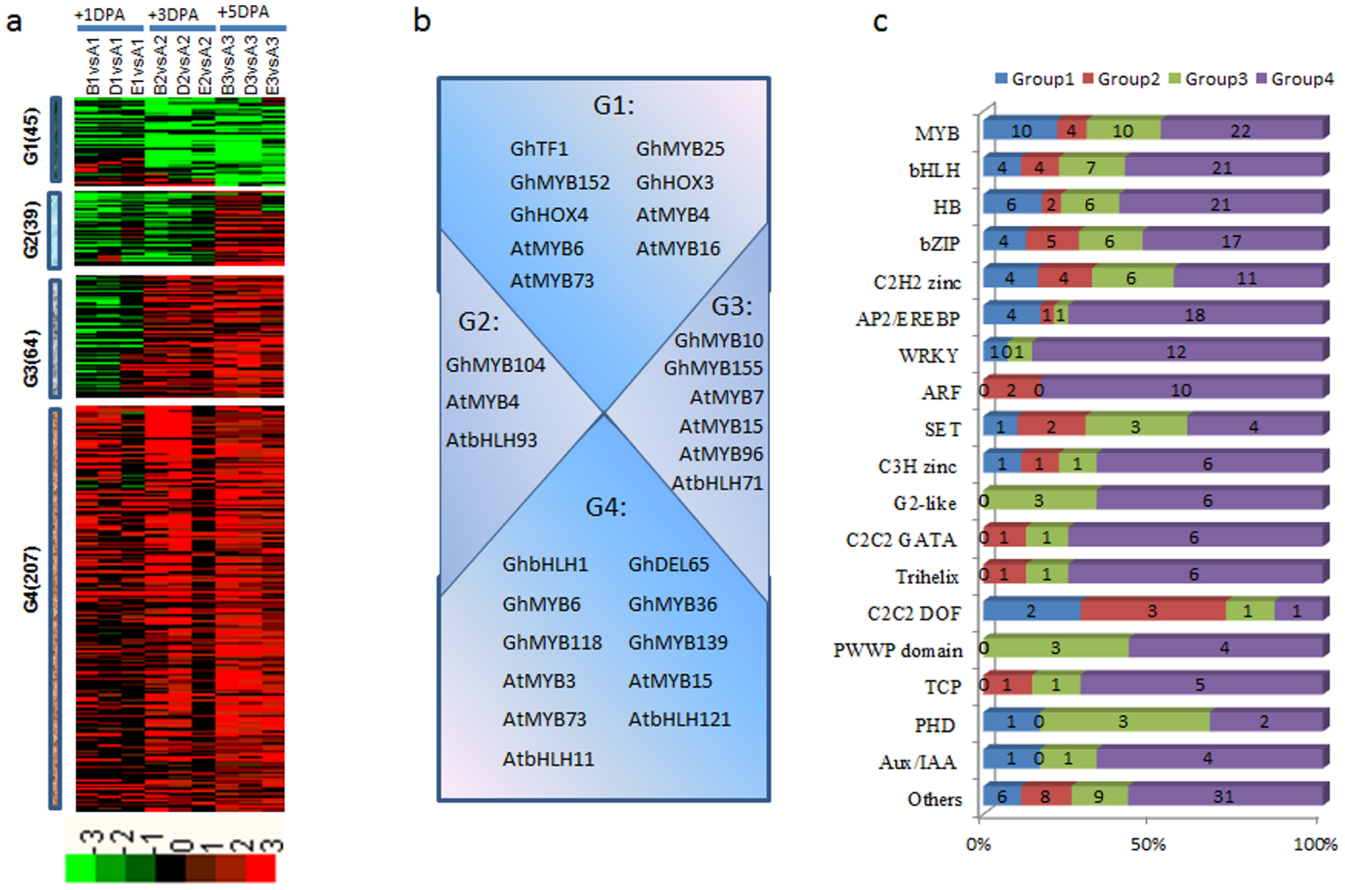
Dynamics of transcription factor expression profiles in various dominant mutants. (a) Unsupervised hierarchical clustering of 355 transcription factor genes included in the 4,358 common DEGs in the dominant mutants. Four groups were generated and the number of each group was in parentheses. Red region, genes upregulated in the mutants; green region, genes downregulated in the mutants. A, TM-1; B, SL1-7-1FLM; D, MD17FLM; E, N_1_NSM; 1, +1 DPA; 2, +3 DPA; 3, +5 DPA. (b) Representative functions and genes showing expression gradients. (c) Distribution of transcription factor families among G1, G2, G3 and G4.

The dynamics of transcription factor accumulation during fuzz initiation were particularly well resolved in our data. Of the 1,596 differentially expressed transcription factor genes that we detected in the fuzz initial stage, 355 were common to the dominant mutants (Figure 3). Most of these genes (207) were upregulated in the dominant mutants (Group4), including *GhbHLH1*, *GhDEL65*, *GhMYB6*, *GhMYB118*, *GhMYB139*, *AtMYB3*, *AtMYB73*, *AtbHLH121* and *AtbHLH11*. Only 45 transcription factors were downregulated (Group 1), including *GhTF1*, *GhMYB25*, *GhMYB152*, *GhHOX3*, *GhHOX4*, *AtMYB4*, *AtMYB6*, *AtMYB16* and *AtMYB73*. Thirty nine transcription factors showed downregulation at +1 DPA and +3 DPA, and upregulation at +5 DPA (Group 2); these included *GhMYB104*, *AtMYB4*, and *AtbHLH93*. An additional 64 transcription factors showed downregulation at +1 DPA, and upregulation at +3 DPA and +5 DPA (Group 3); these included *GhMYB10*, *GhMYB155*, *AtMYB7*, *AtMYB15*, *AtMYB96*, *AtbHLH71*(Figure 3a and 3b). We also identified family-specific expression trends (Figure 3c). Members of the C2C2 DOF zinc-finger families of transcriptional regulators are highly expressed in TM-1; Aux/IAA, WRKY, AP2/EREBP and G2-like families are highly expressed in the dominant mutants.

To identify the metabolic pathways that are active during fuzz initiation, we mapped the 4,358 commonly expressed genes in the dominant mutants to the reference KEGG canonical pathways. In total, we assigned 1448 genes to KEGG pathways. Most of these mapped to ATP synthesis, or sugar and lipid metabolism-related metabolic pathways such as starch and sucrose metabolism (82 members), oxidative phosphorylation (30 members), galactose metabolism (22 members), glycolysis/gluconeogenesis (31 members), fatty acid degradation (20 members; Table S4). These annotations provide a valuable resource for investigating the processes, functions, and pathways specific to the initiation of fuzz development.

### Common DEGs between Recessive Naked-seed Mutants and TM-1 during Fuzz Development

To understand the molecular mechanisms underlying the recessive naked seed phenotype, 6,693 DEGs were identified that are common to mutants XZ142FLM and n_2_NSM compared with TM-1. Of these, 1,978 genes were up-regulated and 1,480 genes downregulated at +1 DPA; 2,971 genes were upregulated and 980 genes downregulated at +3 DPA; and 1,264 genes were upregulated and 666 genes downregulated at +5 DPA. There were 192 upregulated genes and 120 downregulated genes common to the differentially expressed genes at +1 DPA, +3 DPA and +5 DPA (Figure S2, Table S5).

**Figure 4:**
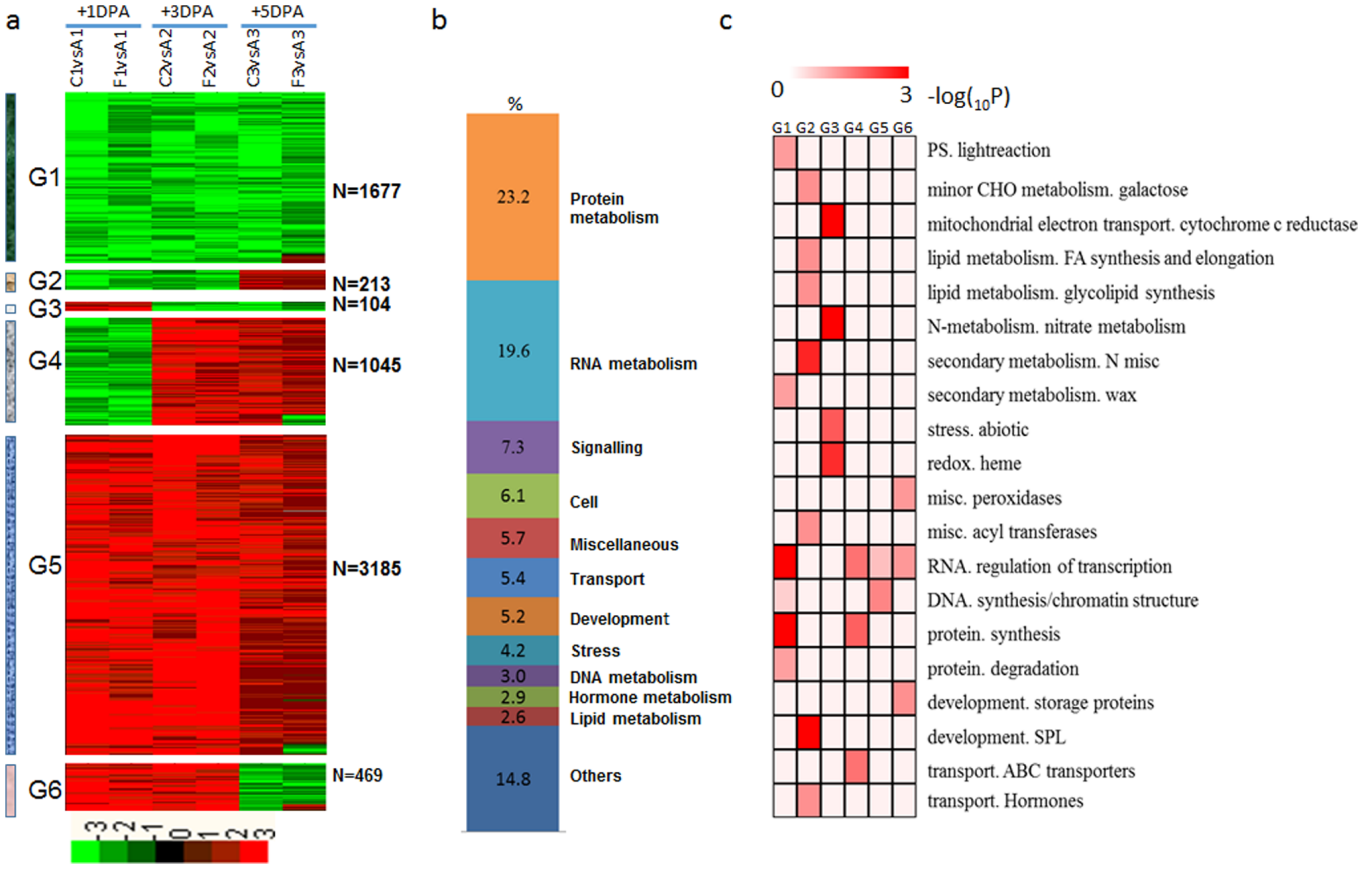
Dynamic progression of common differentially expressed genes in the recessive mutants. (a) Unsupervised hierarchical clustering of 6,693 common differentially expressed genes in the recessive mutants. Common DEGs were clustered into six groups and the number of genes of each group was listed at right. Red region, genes upregulated in the mutants; green region, genes downregulated in the mutants. A, TM-1; C, XZ142FLM; F, n_2_NSM; 1, +1 DPA; 2, +3 DPA; 3, +5 DPA. (b) Functional category distribution of common DEGs in the recessive mutants. (c) Functional category enrichment of common differentially expressed genes in the recessive mutants.

We identified six groups using the hierarchical clustering algorithm. Group 1 and Group 5 accounted for ∼73% of the differentially expressed genes at the three sampled times (approx. 4,852 genes; Figure 4a). Five-thousand four-hundred twenty-one genes had MapMan annotations, excluding 19.0% belonging to the ‘not assigned or unknown’ categories. Among the annotated genes, 23.2% are related to protein metabolism, 19.6% to RNA metabolism, 7.3% to signaling, and the rest to cell functions, transport, development, stress, DNA metabolism, hormone metabolism or lipid metabolism (Figure 4b). Most of the MapMan bins showed enrichment for particular groups (Figure 4c); for example, genes that encode enzymes for protein synthesis and transcription factors in Group 1, SPL protein in Group 2, abiotic stress in Group 3, ABC transport in Group 4, DNA synthesis in Group 5, and peroxidases and storage protein in Group 6.

**Figure 5:**
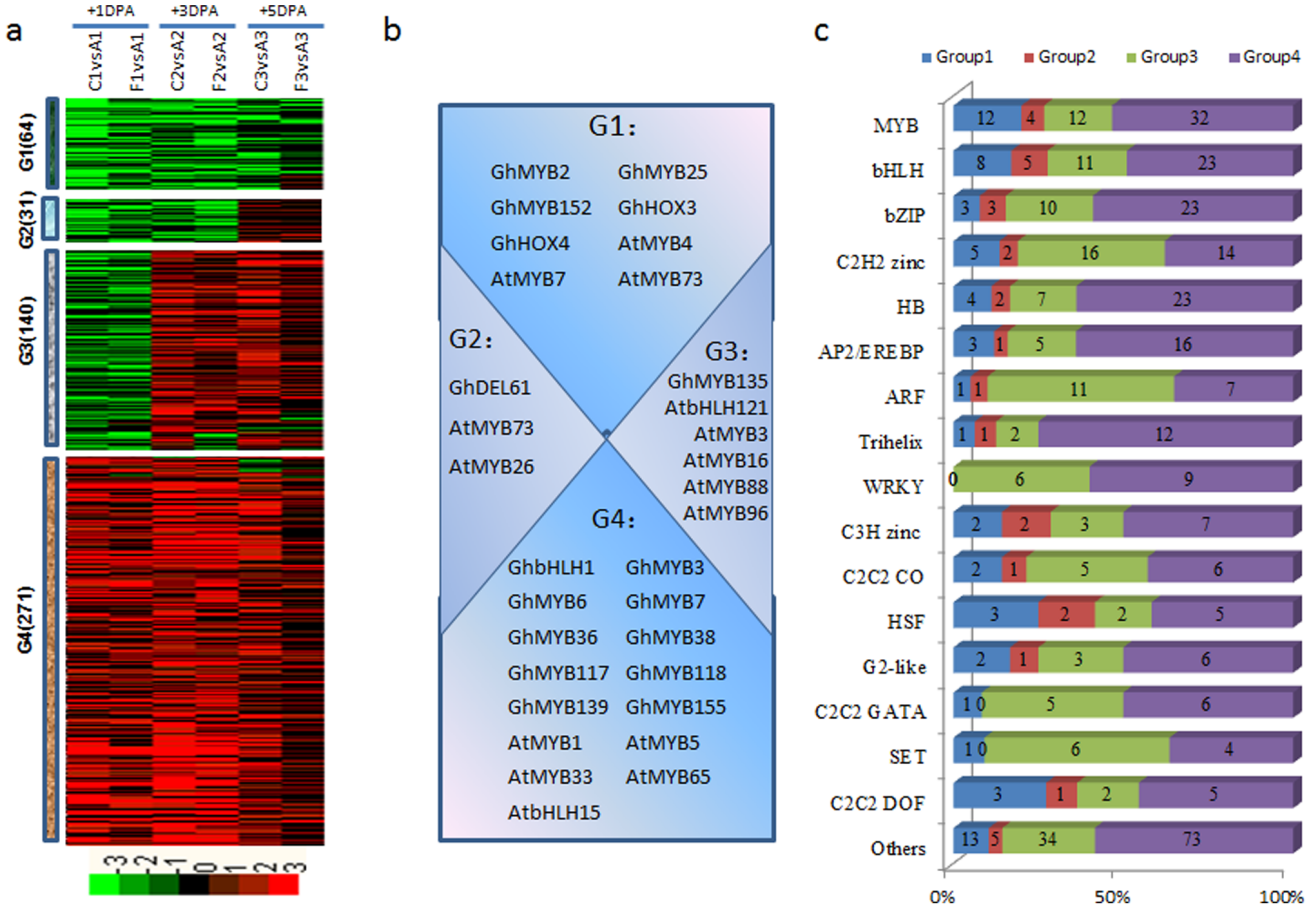
Dynamics of transcription factor accumulation profiles in various recessive mutants. (a) Unsupervised hierarchical clustering of 506 transcription factor genes included in the 6,693 common DEGs in the recessive mutants. Four groups were generated and the number of each group was in parentheses. Red region, genes upregulated in the mutants; green region, genes downregulated in the mutants. A, TM-1; C, XZ142FLM; F, n_2_NSM; 1, +1 DPA; 2, +3 DPA; 3, +5 DPA. (b) Representative functions and genes showing expression gradients. (c) Distribution of transcription factor families among G1, G2, G3 and G4.

We identified 506 transcription factors that were expressed in common in the two recessive mutants (Figure 5). Among these, 64 transcription factors (Group 1) including *GhMYB2*, *GhMYB25*, *GhMYB152*, *GhHOX3*, *GhHOX4* were downregulated in the recessive mutants, and 271 transcription factors (Group4) were upregulated including *GhbHLH1*, *GhMYB3*, *GhMYB36*, *GhMYB7*, *GhMYB36*, *GhMYB38*, *GhMYB117*, *GhMYB118*, *GhMYB139*, *GhMYB155*. Thirty-one transcription factors (Group 2), such as *GhDEL61*, *GhGL2-like1* were downregulated at +1 DPA and +3 DPA, but were upregulated at +5 DPA, 140 transcription factors (Group 3), such as *GhMYB135* were downregulated at +1 DPA, were upregulated at +5 DPA (Figure 5a and 5b). Members of the HSF families of transcriptional regulators were highly expressed in TM-1; the MYB, WRKY, AP2/EREBP, bHLH, ARF and C2C2(Zn) GATA families were highly expressed in the recessive mutants (Figure 5c).

Of 6,693 common DEGs in the recessive mutants, 2,356 were assigned to KEGG pathways. The pathways with the most representation for the unique sequences were ATP synthesis, or sugar and lipid metabolism-related metabolic pathways including starch and sucrose metabolism (123 members), oxidative phosphorylation (44 members), glycolysis/gluconeogenesis (43 members), galactose metabolism (40 members) and fatty acid degradation (28 members) (Table S6).

### Common DEGs between Naked-seed Mutants and TM-1

To uncover shared molecular mechanisms in the dominant and recessive fuzz-less mutants, we identified 1,932 DEGs that were common to the five mutants. Of these, 106 genes were upregulated and 314 downregulated at +1 DPA, 473 genes were upregulated and 215 downregulated at +3 DPA, and 737 genes were upregulated and 432 downregulated at +5 DPA (Figure S2, Table S7). Four were three upregulated genes and 29 downregulated genes common to the +1 DPA, +3 DPA and +5 DPA samples (Table S8).

**Figure 6:**
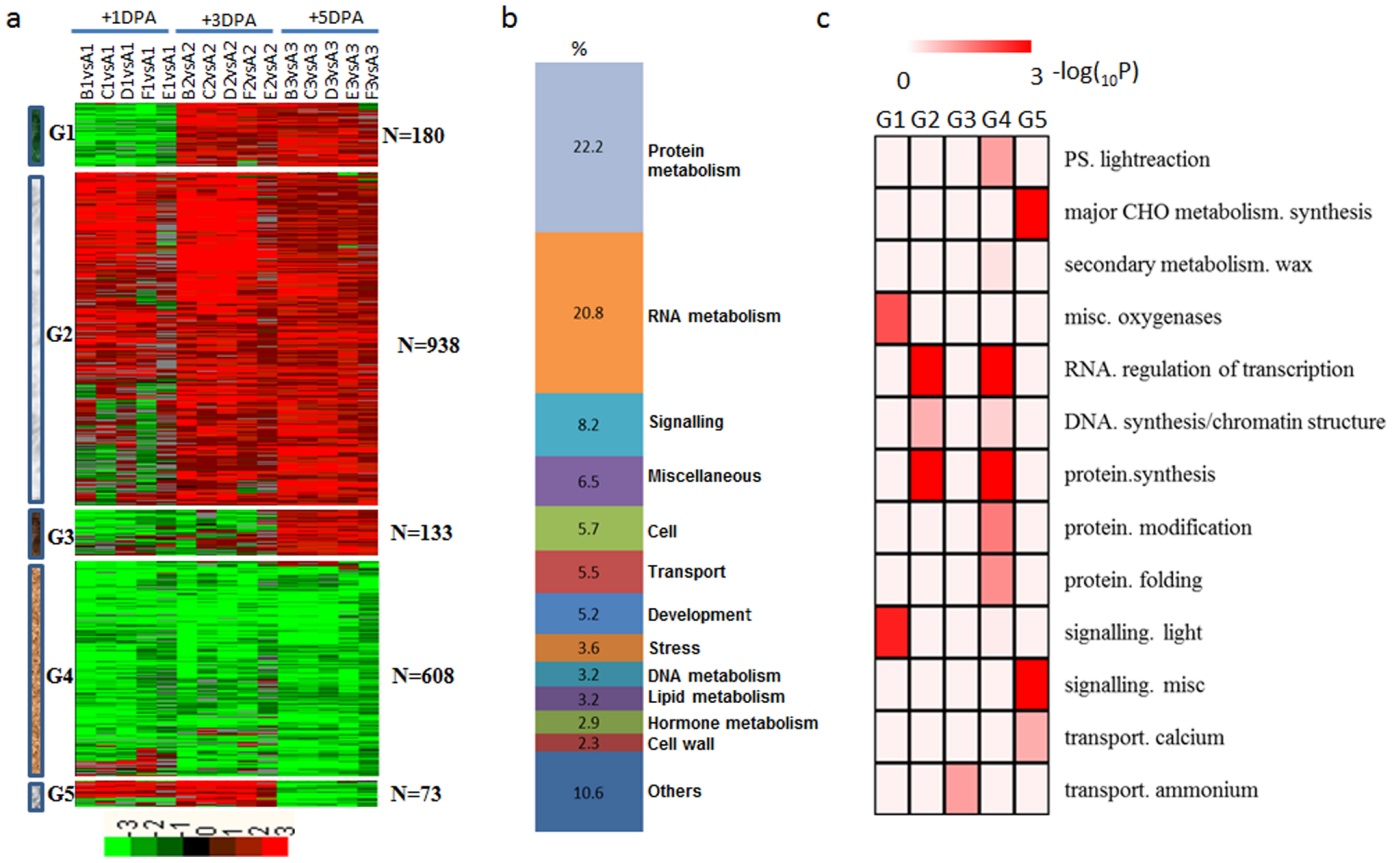
Dynamic progression of common differentially expressed genes in the dominant/recessive mutants. (a) Hierarchical clustering of the 1,932 common DEGs in five mutants. Common DEGs were clustered into five groups and the number of genes of each group was listed at right. Red region, genes upregulated in the mutants; green region, genes downregulated in the mutants. A, TM-1; B, SL1-7-1FLM; C, XZ142FLM; D, MD17FLM; E, N_1_NSM; F, n_2_NSM; 1, +1 DPA; 2, +3 DPA; 3, +5 DPA. (b) Functional distribution of common DEGs in the dominant/recessive mutants. (c) Functional category enrichment of common DEGs in the dominant/recessive mutants.

The 1,932 common DEGs were classified into four groups by hierarchical clustering. Nine-hundred and thirty-eight genes (Group 2) were upregulated at +1 DPA, +3 DPA and +5 DPA; 608 genes (Group 4) were downregulated at +1 DPA, +3 DPA and +5 DPA; 180 genes (Group 1) were downregulated at +1 DPA, and upregulated at +3 DPA and +5 DPA; 133 genes (Group 3) were downregulated at +1 DPA and +3 DPA, and upregulated at +5 DPA; 73 genes (Group 5) were upregulated at +1 DPA and +3 DPA, and downregulated at +5 DPA (Figure 6a).

One-thousand six-hundred and two genes were annotated by MapMan, excluding 17% belonging to the ‘not assigned or unknown’ categories. Among these genes, 22.2% were related to protein metabolism, 20.8% were related to RNA metabolism, 8.2% were related to signaling, with the remaining genes were related to cell functions, transport, development, stress, DNA metabolism, lipid metabolism, hormone metabolism, and cell wall (Figure 6b). Genes that encode oxygenases and light signaling were enriched in Group 1, encode enzymes for protein synthesis and regulation of transcription in Group 2 and 4, ammonium transport in Group 3, and major CHO synthesis in Group 5 (Figure 6c).

**Figure 7:**
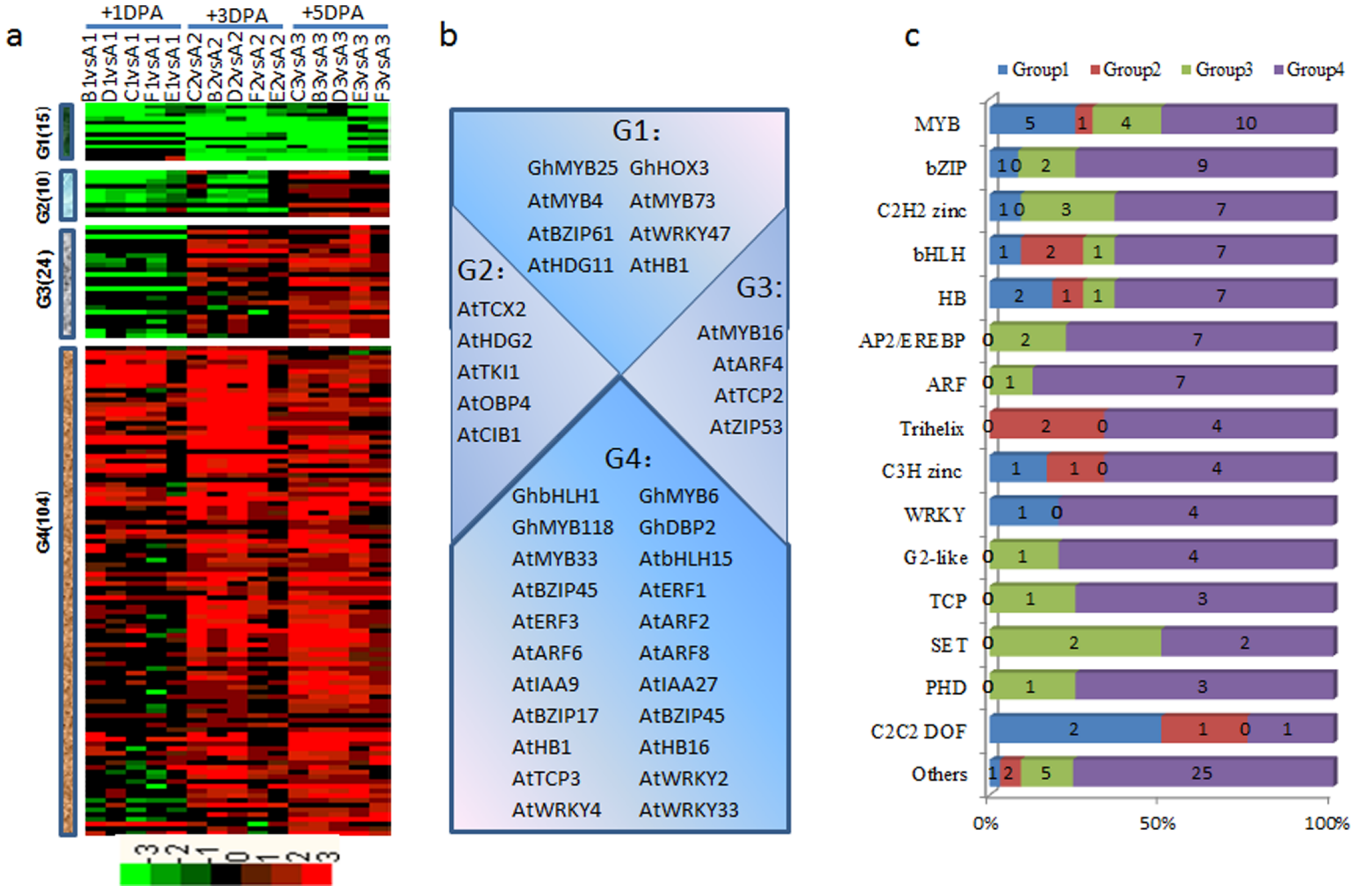
Dynamics of transcription factor accumulation profiles in various dominant/recessive mutants. (a) Unsupervised hierarchical clustering of 153 transcription factor genes included in the 1,932 common DEGs in the recessive mutants. Four groups were generated and the number of each group was in parentheses. Red region, genes upregulated in the mutants; green region, genes downregulated in the mutants. A, TM-1; B, SL1-7-1FLM; C, XZ142FLM; D, MD17FLM; E, N_1_NSM; F, n_2_NSM; 1, +1 DPA; 2, +3 DPA; 3, +5 DPA. (b) Representative functions and genes showing expression gradients. (c) Distribution of transcription factor families among G1, G2, G3 and G4.

We found 153 differentially expressed transcription factor genes in common between the wild-type TM-1 and the naked mutants (Figure 7). Fifteen transcription factors were downregulated in the naked mutants (Group 1), including *GhMYB25 and GhHOX3*. Ten transcription factors were downregulated at +1 DPA and +3 DPA, and upregulated at +5 DPA (Group 2), including *AtTCX2*, *AtHDG2*, *AtTKI1*, *AtOBP4* and *AtCIB1*. Fourty transcription factors were downregulated at +1 DPA, and upregulated at +3 DPA and +5 DPA (Group 3), including *AtMYB16*, *AtARF4*, *AtTCP2*, *AtZIP53*. Another 271 transcription factors showed upregulation in the naked mutants (Group 4), including *GhbHLH1*, *GhMYB6*, *GhMYB118* and *GhDBP2* (Figure 7a and 7b). Members of the most families of transcriptional regulators such as MYB, bHLH, bZIP, C2C2(Zn), HB, AP2/EREBP, ARF and WRKY families were highly expressed in the recessive mutants (Figure 7c).

To identify the differential metabolic pathways active in fuzz initiation, we mapped the 1,932 common DEGs in the dominant and recessive mutants in the KEGG database. In total, we assigned 620 genes to KEGG pathways. Similar to our earlier results, most of these genes were related to ATP synthesis, and sugar and lipid metabolism pathways. For example, 38 genes were annotated to starch and sucrose metabolism 13 genes were annotated to galactose metabolism, and 11 genes to oxidative phosphorylation (Table S9).

### Validation of Differentially Expressed Genes by qPCR

**Figure 8:**
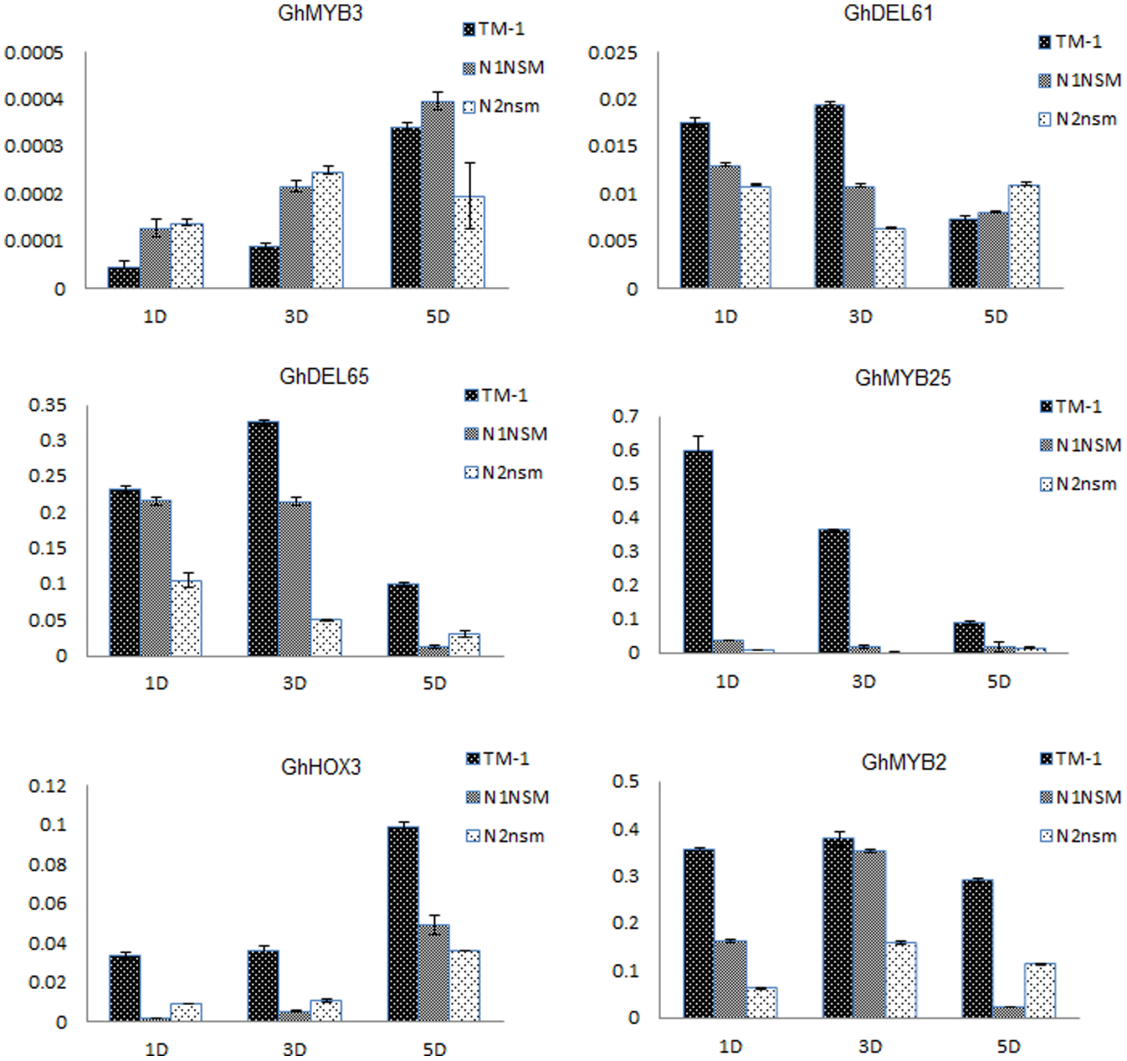
QRT-PCR confirmation for the six selected differentially-expressed transcription factor genes. The expression level of selected genes at +1 DPA, +3 DPA and +5 DPA in TM-1, N_1_NSM and n_2_NSM. Data shown are the means ± SD of three biological replicates.

To determine whether the digital gene expression results were reliable, we conducted qPCR analysis of the expression levels of 21 representative differentially expressed genes, most of them transcription factors. The qPCR results (Table S11) indicated that the expression levels estimated by DGE and qPCR were highly correlated (*r* ^2^ = 0.72–0.93). The qPCR validation results confirmed the accuracy and reliability of the expression levels determined by digital gene expression analysis, which means that we can make reasonable deductions from the functional enrichment analysis of the DEGs. The qPCR results for expression of transcription factors indicated that *GhMYB3* had a high level of expression in fuzzy ovules at +1 DPA and +3 DPA, *GhDEL61* had a low expression level at +1 DPA and +3 DPA, and *GhDEL65* had a low expression level at +3 DPA and +5 DPA. Additionally, *GhMYB25*, *GhHOX3* and *GhMYB2* had low levels of expression in the fuzzless mutants ovules (Figure 8).

## Discussion

### Choice of Materials is Important for the Study of Fuzz Initial Cell Development

Cotton lint fibers are extremely long, single epidermal cells that develop on the outer surface of ovules, reaching upwards of 5 cm in some species [50]. Fibers initiate between −1 DPA and +1 DPA, and the fiber initials begin to elongate rapidly immediately after fertilization, extending out from the surface of the seed coat epidermis. Zhang et al. [1] showed that fuzz initiation begins at +4 DPA by scanning electron microscopy (SEM) examination of TM-1 ovules, although the shape of fuzz protrusions differed from that of lint fibers. In our study, +1, +3 and +5 DPA ovules were employed for fuzz initial development. Two types of fibers, the long lint fibers and the short fuzz fibers, probably share common developmental pathways at least in early differentiation. However, the fuzz fiber appears to be under separate genetic control, as a number of genetic loci specifying absence of fuzz fiber, but with normal lint, have been identified [37]. Lintless mutants, however, only occur in conjunction with lack of fuzz fiber, so are essentially fiberless [49]. Cotton fiber mutants are invaluable for the investigation of genes that control fiber development at the molecular level. The natural fiber mutants are well suited for genetic, physiological, and molecular characterization of the mutant phenotype, avoiding the complex and time-consuming progress of inducing, screening, and verifying fiber mutants. In this study, we selected five mutant lines, all of them fuzzless mutants that possessed different naked-seed genes. Thus, we can more clearly understand the mechanism of regulation of fuzz initial development by studying the five fuzzless mutants.

### Many Specific Proteins that Relate to Fuzz Initial Development were Identified

A global analysis of the transcriptome will facilitate the characterization of gene expression and identification of regulatory mechanisms involved in fiber development [51], [32]. In this study, we performed transcriptome profiling of fuzz-bearing and fuzzless ovules to identify genes that were differentially expressed during the fuzz initiation stage. Using a tag-based deep-sequencing approach [52], we could obtain a direct digital readout of cDNAs and achieve an essentially dynamic range of genes from the libraries. Thus, the present study represents the most comprehensive analyses of the cotton fuzz transcriptome. Approximately 19,829–22,213 tag-mapped reference genes were identified for each library. Unfortunately, sequencing of the upland cotton genome is incomplete, so there are still a large proportion of unique tags mismatched. These unmatched unique sequences probably represent novel genes to be identified in future studies.

From +1 to +5 DPA, the cotton fibers and ovules are in a state of rapid development. Jensen (1968) observed the ultrastructure and composition of the cotton zygote and described a dramatic series of alterations in cell structure including zygote size, endoplasmic reticulum, microtubes, mitochondria, ribosomes and plastids [53]. During early development, fiber cells produced from the surface of the ovules and elongate quickly. We found that DEGs between TM-1 and fuzzless mutants involved in protein metabolism, RNA metabolism, and signaling categories were enriched significantly. The large number of RNA metabolism-related genes is consistent with the sharp increase in the total number of ribosomes observed in the zygote [53].

Based on the large number of genes with fiber-specific expression, the molecular dissection of cotton fiber initiation has provided new insights [33], [34]. Lee et al. identified more than 20 genes that were greatly enriched at the fiber-bearing (+3 DPA) stage in TM-1 as compared to the N_1_NSM mutant [34], [51]. Few studies have been performed to examine the initial pattern of cotton fuzz fiber development. In this study, we identified many DEGs between TM-1 and the fuzzless mutants. Protein synthesis-related genes had low levels of representation in both dominant and recessive mutants, while DNA and chromatin structure-related genes were highly represented. ATP synthesis, and sugar and lipid metabolism-related metabolic pathways play important roles in fuzz initial development. Recently, Du et al. [54] identified proteins related to fuzz fiber initiation in wild-type diploid cotton (*Gossypium arboreum* L.) and its fuzzless mutant by comparative proteomic analysis. They found 71 differentially expressed proteins between diploid Asiatic cotton DPL971 and the fuzzless mutant DPL972, mainly involved in cell response/signal transduction, redox homeostasis, protein metabolism, and energy/carbohydrate metabolism [51]. The differential expression of these proteins demonstrated that rapidly differentiating and expanding fuzz fiber cells experience active protein metabolism [55], [56].

### Fuzz Development May Share Similar Molecular Mechanisms with Leaf Trichome Development in *Arabidopsis*

**Figure 9:**
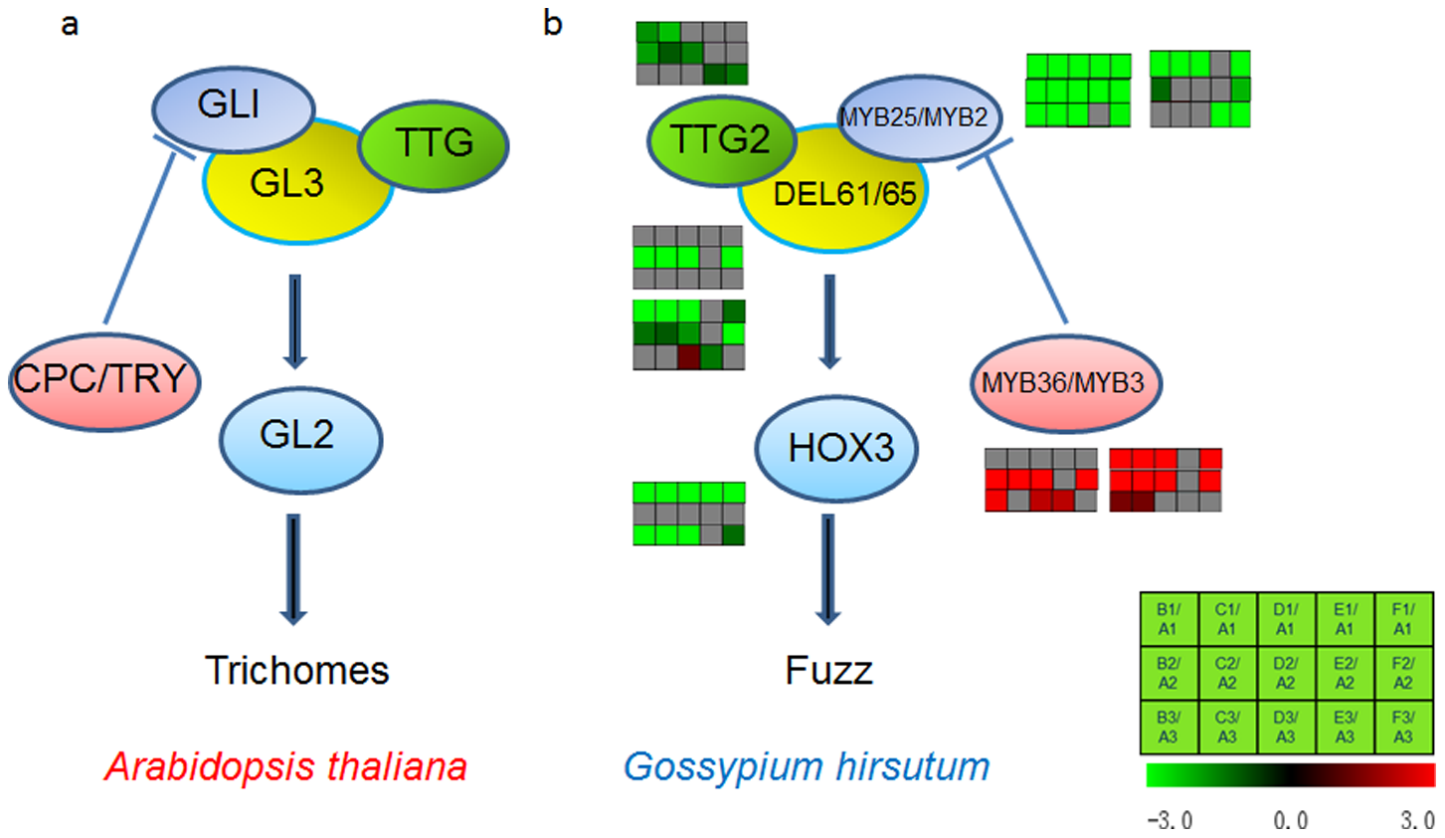
Model for the action of GL1-activating trichome development in *Arabidopsis thaliana* and fuzz development in *Gossypium hirsutum*. a: Model for the action of *GL1*-activating trichome development in *Arabidopsis thaliana.* b: Model for the action of *GL1*-activating fuzz development in *Gossypium hirsutum* A: TM-1, B: SL1-7-1FLM, C: XZ142FLM, D: MD17FLM, E: N_1_NSM, F: n_2_NSM, 1: +1 DPA, 2: +3 DPA, 3: +5 DPA. Light red/green bars indicate cotton fiber gene expression in the upper/lower group.

Illuminating the functions of key regulators in fuzz development could help explain the reasons for the delayed developmental and elongation steps of fuzz fiber development. Through molecular improvement of key transcriptional factors in cultivated varieties, cotton could produce longer fuzz fibers and have higher yields. Cell fate determination is a critical step in the developmental processes of plants, and involves the participation of a large number of transcription factors. The pattern of trichome development has been studied in depth in the model plant *Arabidopsis* [57]. We found many common differentially expressed transcription factors in the dominant and recessive mutants. Most of these were in the MYB, bHLH, HB and WRKY gene families. *GhTTG2*, a putative homolog of *Arabidopsis TTG2*, were downregulated in lintless-fuzzless mutants at +1 DPA and +3 DPA, and also in ovules of lint-fuzzless mutants in +5 DPA. *GhMYB25* and *GhMYB2,* putative homologues of *Arabidopsis GL1*, showed low expression levels in fuzzless mutants. The bHLH domain of *DEL61* and *DEL65*, which share a high degree of sequence identity with *Arabidopsis* GL3 and EGL3, both had low expression levels in the fuzzless mutants. HOX3, a full-length coding sequence homolog of *AtGL2*, shares 72% identity with the homeobox conserved domain, and the expression level of HOX3 in the fuzzless mutants was extremely low compared to that in TM-1. *GhMYB3*/*GhMYB36* pertaining to the MYB family had the high expression level in fuzzy ovules in dominant/recessive mutants (Figure 9). A model of the MYB25/MYB2-DEL61/65-TTG2 protein complex was described as the initial pattern of cotton fuzz, similar to the model of trichomes and root hairs in *Arabidopsis* [33], [53]. Future studies including analyses of protein function may shed light on the mechanism of cell initiation and formation of cotton fiber.

## Supporting Information

Figure S1 **Distribution of clean tag copy numbers for the 18 libraries.**
(TIF)

Figure S2 **Identity analysis of differentially expressed genes in various mutants.**
(TIF)

Table S1 **Genes involed in cotton fiber initial development and their** ***Arabidopsis*** **homologs.**
(XLSX)

Table S2 **Raw sequence data for the 18 transcriptome libraries.**
(XLSX)

Table S3 **List of 4,358 common DEGs between the mutants MD17FLM, SL1-7-1FLM, N1NSM and the wild-type TM-1.**
(XLS)

Table S4 **Pathway enrichment analysis for common differentially expressed genes in various dominant mutants.**
(XLS)

Table S5 **List of 6,693 common DEGs between the mutants XZ142FLM and n2NSM and the wild-type TM-1.**
(XLS)

Table S6 **Pathway enrichment analysis for common differentially expressed genes in various recessive mutants.**
(XLS)

Table S7 **List of 1,932 common DEGs between the mutants MD17FLM, SL1-7-1FLM, N1NSM, XZ142FLM, n2NSM and the wild-type TM-1.**
(XLS)

Table S8 **The common differentially expressed genes in various mutants and times.**
(XLS)

Table S9 **Pathway enrichment analysis for common differentially expressed genes in various dominant and recessive mutants.**
(XLS)

Table S10 **Primers used for qRT-PCR analysis.**
(XLS)

Table S11 **Correlation between qRT-PCR and DGE for 21 differentially expressed genes.**
(XLS)
