# Supplementary figures and images for: Genome-Wide Transcriptome Profiling Revealed Cotton Fuzz Fiber Development Having a Similar Molecular Model as Arabidopsis Trichome
Source: PLoS One. 2014 May 13;9(5):e97313. doi: 10.1371/journal.pone.0097313 (PMC4019585; doi:10.1371/journal.pone.0097313)

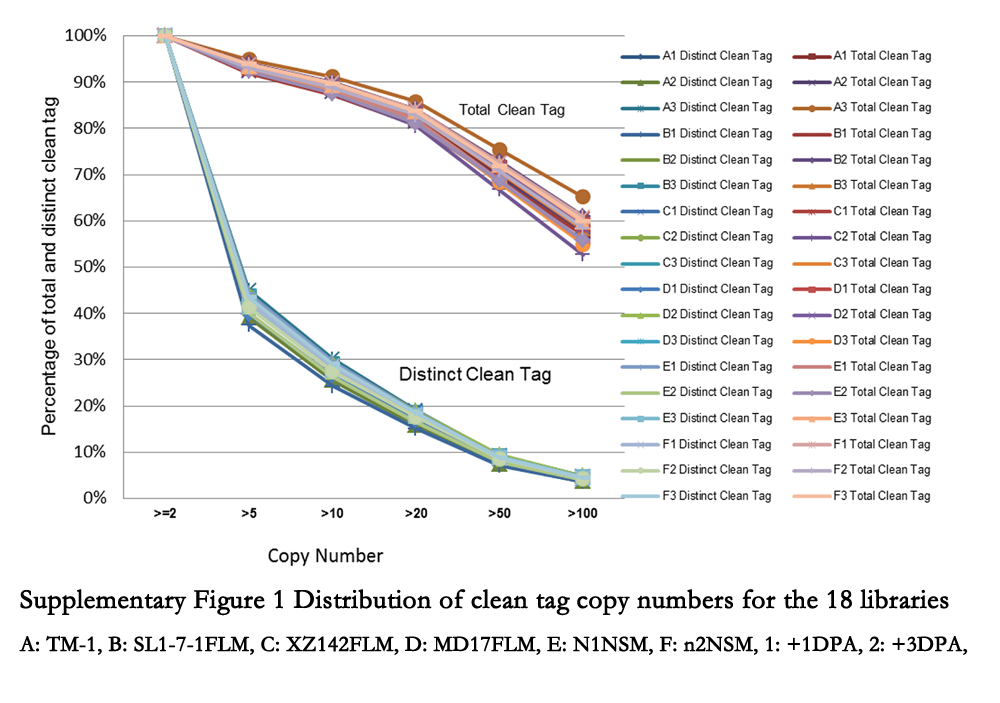

Supplement: Figure S1 — Distribution of clean tag copy numbers for the 18 libraries. (TIF) [file pone.0097313.s001.tif]

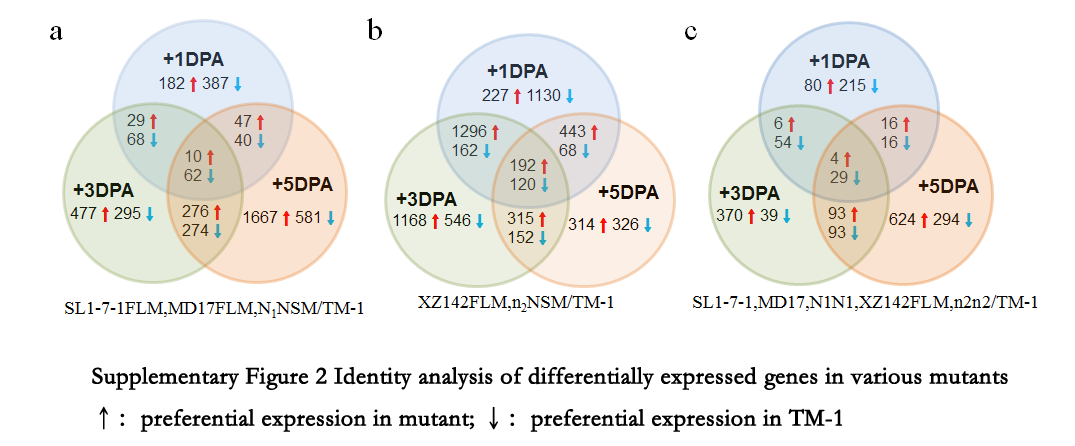

Supplement: Figure S2 — Identity analysis of differentially expressed genes in various mutants. (TIF) [file pone.0097313.s002.tif]
